# Efficient Data Aggregation in Smart Grids: A Personalized Local Differential Privacy Scheme

**DOI:** 10.3390/s26051710

**Published:** 2026-03-08

**Authors:** Haina Song, Jinhang Sun, Mengyao Wang, Nan Zhao, Fan Zhang, Hongzhang Liu

**Affiliations:** 1 Hubei Key Laboratory for High-Efficiency Utilization of Solar Energy and Operation Control of Energy Storage System, Hubei University of Technology, Wuhan 430068, China; songhn_cqupt@163.com (H.S.); 19972916126@163.com (J.S.); wangmy19888@163.com (M.W.); nzhao@mail.hbut.edu.cn (N.Z.); joyce_zhang@hbut.edu.cn (F.Z.); 2 School of Nuclear Technology and Chemistry & Biology, Hubei University of Science and Technology, Xianning 437100, China

**Keywords:** smart grid, local differential privacy, personalized privacy protection, data aggregation

## Abstract

The rapid advancement of smart grids, while enhancing the efficiency of power systems, has also raised serious concerns regarding the privacy and security of end-users’ electricity consumption data. Traditional privacy protection methods struggle to meet users’ individualized privacy requirements and often lead to a significant decline in data aggregation accuracy. To address the core contradiction between personalized privacy protection and high-precision grid analytics, this paper proposes an efficient data aggregation scheme based on personalized local differential privacy (EDAS-PLDP) tailored for smart grids. The proposed scheme enables smart terminal users to autonomously select their privacy protection levels based on individual needs, thereby breaking the limitations of the traditional “one-size-fits-all” approach. To mitigate the accuracy loss caused by personalized perturbations, a mean square error-based weighted aggregation strategy is introduced at the gateway side. This strategy evaluates the data quality from groups with different privacy preferences and adjusts aggregation weights to optimize the estimation accuracy of the global mean electricity consumption. Extensive experimental results demonstrate that, compared to existing mainstream schemes, EDAS-PLDP achieves higher estimation accuracy under various distributions of privacy preferences, user scales, and data granularities, while exhibiting lower time consumption, making it suitable for resource-constrained smart grid environments. Furthermore, the scheme shows excellent robustness against false data injection attacks. In summary, EDAS-PLDP provides a balanced and efficient solution for reconciling personalized privacy protection with high-precision data utility in smart grids.

## 1. Introduction

As an innovative evolution of modern power systems, smart grids [1] are characterized by the profound convergence of information technology and the electric power industry, enabling intelligent monitoring, efficient management, and precise dispatching [2,3,4]. However, their widespread application also raises serious challenges to data privacy and security [5,6].

In smart grids, vast numbers of smart meters continuously collect end-users’ real-time electricity consumption data, revealing detailed information about usage behaviors and appliance operation patterns [7,8]. Leakage of such data could expose private information—such as daily habits—to unauthorized parties [9], intruding on privacy and potentially causing economic losses. Critically, different end-users exhibit divergent privacy demands: some are highly sensitive and willing to trade accuracy for stronger guarantees, while others prioritize data utility over stringent protection. However, traditional privacy mechanisms in smart grids often adopt a “one-size-fits-all” approach, failing to accommodate such personalized needs [10].

As electricity data scales and refines, balancing personalized privacy protection with high data utility has become a critical challenge. Personalized local differential privacy offers a promising approach for decentralized protection, yet its generic mechanisms often fail to adequately address the dual constraints of aggregation accuracy and terminal computing power in smart grid scenarios. Therefore, a personalized privacy-preserving framework tailored for smart grids is needed—one that can harmonize individualized privacy requirements, data utility, and system efficiency.

In the interdisciplinary field of computer science and power systems, various data privacy protection techniques have been proposed and applied for smart grids. Nevertheless, existing solutions still face significant limitations when deeply integrated into grid operational scenarios. For instance, while techniques such as homomorphic encryption [11] and data anonymization [12] can offer a certain degree of privacy protection for smart meter data, homomorphic encryption typically incurs high computational overhead, making it unsuitable for deployment on resource-constrained grid devices, whereas anonymization is often susceptible to background knowledge attacks. These shortcomings consequently undermine their effectiveness in meeting the smart grid’s requirements for accurate data analysis and practical utility. Some centralized differential privacy approaches [13,14] can provide strong privacy guarantees, but their dependence on a trusted third party introduces inherent risks: in complex network environments, achieving a fully trusted third party is challenging, and once such a party is compromised, end-user data faces a severe risk of leakage.

Based on the above considerations, Gai et al. [15] proposed a smart grid data aggregation scheme based on local differential privacy (LDP), which employs a randomized response mechanism based on K-RR for the aggregated collection of electricity consumption data. However, a primary limitation of this approach lies in its assignment of a uniform privacy budget to all end-users, failing to accommodate individualized privacy protection needs. He et al. [16] proposed a lightweight multi-dimensional data aggregation scheme based on the Chinese Remainder Theorem, which significantly reduces communication overhead through compression techniques but still employs globally uniform privacy parameters. Zhang et al. [17] innovatively adopted a data-domain grouping perturbation strategy, where high-precision electricity consumption data is divided into digit-based groups and perturbed separately, effectively controlling computational costs, yet it similarly lacks support for personalized privacy requirements. Shanmugarasa et al. [18] proposed a LDP method based on a sliding window, specifically designed to protect device-level energy consumption temporal data during sharing. However, this method does not incorporate a personalization mechanism nor optimize its accuracy at the grid aggregation level. To address user-specific privacy needs, Li et al. [19] proposed a personalized local differential privacy (PLDP) data collection scheme, allowing end-users to freely select privacy protection levels and corresponding perturbation methods based on their individual requirements. Nevertheless, this scheme suffers from high computational complexity during both the perturbation and aggregation phases, and its aggregation accuracy remains unsatisfactory.

In summary, existing schemes have yet to achieve an ideal balance among personalized privacy support, aggregation accuracy, and system overhead: some schemes lack personalization mechanisms and cannot meet users’ diverse privacy needs; while others support personalization, they sacrifice data utility or introduce high computational costs. Therefore, how to accommodate users’ diverse privacy requirements while ensuring high accuracy and low overhead remains a critical challenge urgently needing resolution in the field of smart grid privacy protection. To address the aforementioned challenges, this paper proposes an efficient data aggregation scheme based on personalized local differential privacy (EDAS-PLDP). This scheme aims to integrate personalized local differential privacy by enabling end-users to autonomously select protection levels that align with their varying privacy requirements, and leverages a Mean Square Error-based weighted aggregation framework to dynamically integrate perturbed data from different privacy subgroups, thereby enhancing aggregation accuracy. The main contributions of this paper are summarized as follows:

1. To address end-users’ personalized privacy protection requirements in smart grids, we propose a terminal data perturbation mechanism based on PLDP, which is designed to allow end-users to autonomously select privacy protection levels. This design enables end-users with varying privacy needs to flexibly balance privacy and data utility, breaking the limitations of the traditional “one-size-fits-all” approach.
2. To meet the demand for high data accuracy in grid load analysis, a novel weighted aggregation strategy based on mean square error is introduced. This strategy enables the gateway to effectively assess the data quality of different privacy subgroups and assign corresponding aggregation weights when integrating localized electricity consumption data from various privacy cohorts, significantly improving the estimation accuracy of the global mean electricity consumption compared to existing methods.
3. The simulation results demonstrate that the proposed EDAS-PLDP scheme outperforms existing methods across multiple dimensions. In terms of accuracy, compared to baseline schemes, EDAS-PLDP reduces the mean square error by at least 34% under various privacy preference distributions, user scales, and data granularities. In terms of efficiency, terminal perturbation requires only $1.3\times 10^{-5}$ s, and gateway-side aggregation for one million users can be completed within 2 s, significantly outperforming Li’s scheme. In terms of security, it exhibits stronger robustness against false data injection attacks.
4. From a practical deployment perspective, EDAS-PLDP exhibits excellent scalability. Its lightweight design enables deployment on resource-constrained smart meter terminals, while the efficient aggregation mechanism on the gateway side can support smart grid application scenarios with millions of users, providing a feasible pathway for the implementation of personalized privacy protection technologies in real-world grid environments.

## 2. Smart Grid and Privacy Security

This section begins by outlining the typical network architecture of a smart grid, which serves as the infrastructure for data collection and aggregation. Subsequently, we analyze the main privacy threats faced by electricity consumption data in such environments.

### 2.1. Intelligent Grid Network Architecture

The network architecture of the smart grid typically comprises three primary layers [20]: the smart meter (SM), the aggregation gateway (AG), and the control center (CC). The functions of each layer are described below:

- SM: Each end-user is equipped with a smart terminal. During operation, the SM periodically collects the end-user’s electricity consumption data and locally perturbs these data via a randomized response mechanism.
- AG: Each AG acts as a data aggregator in the smart grid. It is responsible for collecting and summarizing the electricity consumption data from all SMs in its designated area. Before transmitting the data to the CC, the AG performs preliminary analysis and validation on the aggregated dataset, including the calculation of key indicators such as average electricity consumption and total usage per region.
- CC: The CC connects to all AGs and aggregates the information received from them. It conducts in-depth analytical processing on the collected data. Based on the results, the CC formulates targeted, data-driven strategies for grid adjustment to enhance overall operational efficiency.

### 2.2. Privacy Attack Model in Smart Grids

In smart grids, users’ electricity consumption data face various privacy attack threats during collection, transmission, and aggregation, primarily manifested as differential attacks and false data injection attacks. Differential attacks infer individuals’ sensitive information by analyzing changes in statistical characteristics of data before and after perturbation. For instance, by comparing the total electricity consumption of a residential area on two different days—one including the target user and one excluding them—an attacker can deduce the user’s precise usage and, over time, infer their daily routines, potentially enabling crimes such as burglary. False data injection involves forging or tampering with electricity consumption data uploaded by smart meters (e.g., injecting abnormally high or low consumption values) with the intention of disrupting grid operations. For example, attackers may inject false high consumption data to simulate a sudden demand peak in a certain area, causing the load forecasting system to misjudge a power shortage and trigger unnecessary emergency dispatches, resulting in resource waste. To address these threats, the proposed scheme provides protection through the following measures:

1. The proposed EDAS-PLDP scheme employs a probability perturbation mechanism based on interval design, which ensures that there is no reversible mapping relationship between any single real data input and its corresponding noisy output, while strictly satisfying the mathematical definition of local differential privacy. This mechanism, through a bounded randomization process, ensures that even with maximal background knowledge, an attacker cannot infer the original information of a specific user from the perturbed data, thereby effectively defending against differential attacks.
2. The proposed scheme exhibits inherent robustness against false data injection attacks due to its distributed perturbation architecture. Since each smart meter performs data perturbation locally, attackers cannot directly manipulate the data distribution at the aggregation stage. Moreover, the mean square error-based weighted aggregation strategy continuously evaluates the credibility of data from different privacy groups. If false data are injected, the degradation in data quality for the affected group—manifested as an increase in mean square error—results in an automatic reduction of its assigned weight during global aggregation, thereby minimizing the impact of the falsified data on the final results.

## 3. Personalized Local Differential Privacy

LDP is designed for scenarios involving untrusted third parties, empowering end-users to locally process and perturb their sensitive personal data, thereby providing a more rigorous privacy guarantee. Unlike centralized differential privacy, which relies on the assumption of a trusted data collector, LDP is tailored for environments with untrusted collectors and requires no additional trusted servers. Its definition is as follows:

**Definition** **1:** *A randomized algorithm* M *satisfies* ε *-LDP if and only if for any pair of records* x *and* $x^{\prime }$ *, and for all possible outputs* y *, the following condition holds [21]:*

(1) $$\mathrm{Pr}[M(x)=y]\leq e^{\varepsilon }\times \mathrm{Pr}[M(x^{\prime })=y]$$

Here, ε represents the privacy budget, where a smaller value indicates a higher level of privacy protection.

Traditional LDP mechanisms do not account for the personalized privacy preferences of local end-users, resulting in either insufficient protection or over-protection. Consequently, under the PLDP framework, each local end-user can select a different privacy budget $\varepsilon_{i}$ according to their own privacy protection requirements, thereby providing personalized privacy protection. The mathematical definition is as follows:

**Definition** **2:** *A randomized mechanism* M *satisfies* $\varepsilon_{i}$ *-PLDP [22] if and only if for any two inputs* t *and* $t^{\prime }$ *, the personalized privacy budget* $\varepsilon_{i}$ *of end-user* u *, and any possible output* z *, the following condition holds:*

(2) $$\frac{\mathrm{Pr}(M(t)=z)}{\mathrm{Pr}(M(t^{\prime })=z)}\leq e^{\varepsilon_{i}}$$

The PLDP allows for a more flexible trade-off between privacy protection and data utility [23], accommodating the personalized privacy requirements of different end-users.

## 4. Personalized Privacy Protection Schemes in Smart Grids

Building upon the theoretical foundations of personalized local differential privacy, this section introduces our proposed EDAS-PLDP scheme tailored for smart grid environments. We first provide a high-level overview of the scheme’s workflow and core components, followed by detailed explanations of its initialization, perturbation, and aggregation mechanisms.

### 4.1. Scheme Overview

**Figure 1:**
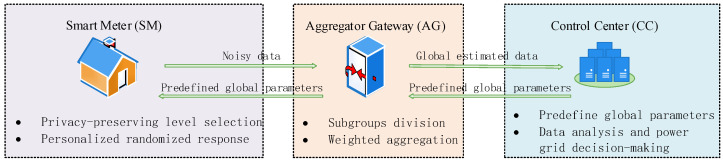
The model framework of our EDAS-PLDP.

In a smart grid environment, the statistical analysis of end-users’ electricity consumption data within a specific region inevitably risks privacy leakage if raw data are collected directly. To address this issue, the proposed EDAS-PLDP leverages the typical three-layer smart grid architecture—SM, AG, and CC—and adopts a hierarchical collaborative mechanism to jointly optimize privacy protection and data aggregation. Figure 1 illustrates the overall workflow of the EDAS-PLDP scheme, with the main steps outlined below:

- Predefine global parameters and definitions at the CC. The CC first predefines m privacy-preserving levels and their corresponding privacy budget parameters. Suppose the electricity consumption data domain is partitioned into *d* subintervals, yielding *k* boundary values (*k* = *d* + 1). The CC broadcasts these predefined global parameters and definitions to all SMs.
- End-user side privacy-preserving level selection at the SM. Each SM captures its true electricity consumption data and then freely determines its privacy-preserving level according to its individual privacy requirement.
- Subgroups division at the AG. The AG groups all end-users into m subgroups based on their chosen privacy-preserving levels. In such a case, end-users with the same privacy-preserving level are grouped into the same subgroup.
- Personalized randomized response at the SM. Each end-user first discretizes its electricity consumption data and then perturbs its private data using a personalized RR mechanism that respects the corresponding privacy budget. The perturbed results are then uploaded to the AG.
- Weighted aggregation at the AG. The AG aggregates the perturbed data from all end-users, performs statistical analysis, and constructs a mean square error-based weighted aggregation strategy to obtain a more accurate global estimate. This global estimate serves as input for downstream smart grid decision-making.

Through this layered design—where global parameters are set at the CC, the privacy-preserving levels are selected by end-users at the SM, and weighted aggregation is performed at the AG—our EDAS-PLDP achieves personalized privacy preservation while substantially improving the utility of the aggregated data.

### 4.2. System Initialization

During the system initialization phase, the CC predefines m privacy-preserving levels $L_{1},L_{2},\ldots ,L_{m}$. The range of electricity consumption data, [0,I], is divided into d subintervals: $\left\{(0,s),(s,2s),\ldots ,\left[(d-1)s,ds\right]\right\}$, where $d=\left\lceil I/s\right\rceil$. The AG can assign a specific value to s according to the actual electricity usage conditions. The set of boundary values for these d subintervals is denoted as $D=\left\{d_{1},d_{2},d_{3},\ldots ,d_{k}\right\}$. The CC distributes the above parameters to all SMs. The end-user freely selects a privacy-preserving level $L_{\tau }$ according to its own privacy requirements. The AG categorizes all end-users into m subgroups $G_{1},G_{2},\ldots ,G_{m}$ based on their selections. The end-users with the same privacy-preserving level are assigned to the same subgroup.

Take the τ-th subgroup $G_{\tau }$ as an example, and let $x_{\tau i}$ denote the raw data of the i-th end-user in $G_{\tau }$. Since electricity consumption data in smart grids are continuous numerical values, directly applying traditional categorical perturbation mechanisms may lead to a sharp decline in utility due to the large value range. Therefore, before applying the personalized randomized response perturbation, the SM first discretizes $x_{\tau i}$ into the specific boundary. The data discretization is given in Algorithm 1, and the discretization procedure is as follows:

- Step 1: Determine the electricity consumption subinterval to which the end-user’s data belongs. The SM identifies the subinterval corresponding to $x_{\tau i}$. For ease of describing the discretization process, this subinterval is denoted as $\left[u,v\right]$, where $u=\left\lfloor x_{\tau i}/s\right\rfloor \cdot s$ and $v=\left(\left\lfloor x_{\tau i}/s\right\rfloor +1\right)\cdot s$.
- Step 2: Discretize the private data based on conditional-probability. Each end-user adopts the following conditional-probability rule to map the raw private value $x_{\tau i}$ into a discrete value $x_{\tau i}^{\prime }$. The discretization strategy is as follows:
  (3) $$\mathrm{Pr}\left(x_{\tau i}^{\prime }|x_{\tau i}\right)=\left\{\begin{array}{ll} \frac{v-x_{\tau i}}{s}, & x_{\tau i}^{\prime }=u\\ \frac{x_{\tau i}-u}{s}, & x_{\tau i}^{\prime }=v \end{array}\right.$$

Thus, $x_{\tau i}$ is discretized into either the left boundary value u or the right boundary value v of its corresponding electricity consumption subinterval $\left[u,v\right]$ based on the conditional probability $\mathrm{Pr}=\left(x_{\tau i}^{\prime }|x_{\tau i}\right)$. Subsequently, the SM locally perturbs the $n_{\tau }$ discrete data points $X_{\tau }^{\prime }=\left\{x_{\tau 1}^{\prime },x_{\tau 2}^{\prime },\ldots ,x_{\tau n_{\tau }}^{\prime }\right\}$.
**Algorithm 1.** Data Discretization**Input**: Raw electricity consumption data $x_{\tau i}$, boundary set $D=\left\{0,s,2s,\ldots ,ds\right\}$ **Output:** Discretized data $x_{\tau i}^{\prime }$
1. Determine the electricity consumption subinterval to which the data belongs $\left[u,v\right]$: 
    $u=\left\lfloor x_{\tau i}/s\right\rfloor \cdot s$, $v=\left(\left\lfloor x_{\tau i}/s\right\rfloor +1\right)\cdot s$
2. Compute the conditional probability based on the raw data $x_{\tau i}$: 
    $\mathrm{Pr}\left(x_{\tau i}^{\prime }|x_{\tau i}\right)=\left\{\begin{array}{ll} \frac{v-x_{\tau i}}{s}, & x_{\tau i}^{\prime }=u\\ \frac{x_{\tau i}-u}{s}, & x_{\tau i}^{\prime }=v \end{array}\right.$
3. Discretize $x_{\tau i}$ into $x_{\tau i}^{\prime }$ by the conditional probability: 
    $p_{u}=\frac{v-x_{\tau i}}{s}$

    if rand(1) < $p_{u}$ then 
        $x_{\tau i}^{\prime }=u$

    else 
        $x_{\tau i}^{\prime }=v$

    end if
4. Return $x_{\tau i}^{\prime }$

### 4.3. Data Perturbation via Personalized Random Response

In smart grid data collection, our proposed scheme adopts a personalized randomized response mechanism to perturb discretized end-user electricity consumption data, thereby fulfilling the requirements of personalized local differential privacy. The core principle of this mechanism is to protect privacy via randomized response, while allowing end-users’ personalized privacy preferences to directly shape the perturbation strategy.

As previously mentioned, the smart grid system predefines m privacy-preserving levels, where the τ-th privacy-preserving level $L_{\tau }$ corresponds to the privacy budget $\varepsilon_{\tau }$ ($\tau \in \left\{1,2,\ldots ,m\right\}$): a larger privacy budget $\varepsilon_{\tau }$ implies a weaker privacy protection preference and a higher data utility; conversely, the opposite holds.

For the i-th end-user in subgroup $G_{\tau }$, its discretized data is denoted by $x_{\tau i}^{\prime }$. Its corresponding perturbed data after perturbation is denoted by $y_{\tau i}\in D$. To satisfy the $\varepsilon_{\tau }$-LDP requirements defined in Section 3, each SM must perturb its discretized data $x_{\tau i}^{\prime }$ before transmission. This paper adopts a personalized randomized response mechanism, with the perturbation probability designed as follows:

(4) $$\mathrm{Pr}(y_{\tau i}|x_{\tau i}^{\prime })=\left\{\begin{array}{ll} p_{\tau }=\frac{e^{\varepsilon_{\tau }}}{|D|-1+e^{\varepsilon_{\tau }}}, & y_{\tau i}=x_{\tau i}^{\prime }\\ q_{\tau }=\frac{1}{|D|-1+e^{\varepsilon_{\tau }}}, & y_{\tau i}\neq x_{\tau i}^{\prime } \end{array}\right.$$

where $D=\left\{d_{1},d_{2},d_{3},\ldots ,d_{k}\right\}$ denotes the set of boundary values after discretization, |D| denotes the total number of discretized boundary values. During the perturbation process, the discrete electricity consumption data $x_{\tau i}^{\prime }$ is output directly as the true value $x_{\tau i}^{\prime }$ with probability $p_{\tau }$, and with probability $q_{\tau }$, it is perturbed into any boundary value from the set D (which contains all boundary values except $x_{\tau i}^{\prime }$) as the perturbed electricity consumption data. Algorithm 2 presents the personalized randomized perturbation algorithm for end-users in subgroup $G_{\tau }$.
**Algorithm 2.** Personalized Randomized Response**Input:** Discretized data $x_{\tau i}^{\prime }$, privacy budget $\varepsilon_{\tau }$, boundary set $D=\left\{d_{1},d_{2},d_{3},\ldots ,d_{k}\right\}$
**Output:** Perturbed data $y_{\tau i}$
1. Calculate the perturbation probability: $p_{\tau }=e^{\varepsilon_{\tau }}/\left(|D|-1+e^{\varepsilon_{\tau }}\right)$
2. if rand(1) < $p_{\tau }$ then
3.        $y_{\tau i}\leftarrow x_{\tau i}^{\prime }$
4.   else
5.        $D^{*}=\left\{d_{i}|d_{i}\in D,d_{i}\neq x_{\tau i}^{\prime }\right\}$;
6.        $y_{\tau i}\leftarrow$ Randomly select a tuple from $D^{*}$;
7.   end if
8. Return $y_{\tau i}$

### 4.4. Data Aggregation Using Weighted Aggregation

After performing personalized perturbation at the terminal level, the subsequent challenge lies in accurately aggregating the perturbed data while preserving utility. This subsection elaborates on the weighted aggregation strategy designed to address this challenge, beginning with the aggregation of data within each privacy subgroup.

#### 4.4.1. Aggregation of Subgroups’ Data

Because the privacy budget varies with the privacy-preserving level, the estimation cannot be performed jointly across different privacy-preserving levels; instead, it must be carried out separately for each privacy-preserving level. Below, we analyze the estimated local mean of the electricity-consumption data for the subgroup $G_{\tau }$.

All end-users in subgroup $G_{\tau }$ first discretize the private data and then perturb them using the personalized randomized response. Then, the subgroup $G_{\tau }$ will obtain $n_{\tau }$ perturbed results defined as $Y_{\tau }=\left\{y_{\tau 1},y_{\tau 2},\ldots ,y_{\tau n_{\tau }}\right\}$. Let $\theta_{\tau }(d_{j})$ denote the number of the *j*-th discretized boundary value $d_{j}(j\in \left\{1,2,\ldots ,k\right\})$ in the noisy data $Y_{\tau }$. Define $\phi_{\tau j}$ as the true frequency of the *j*-th discretized boundary value $d_{j}$ in subgroup $G_{\tau }$, and ${\overset{\wedge }{\phi }}_{\tau j}$ be its corresponding estimated frequency. The AG applies an unbiased estimation to eliminate the bias introduced by the randomized response perturbation in the subgroup $G_{\tau }$ given by

(5) $${\overset{\wedge }{\phi }}_{\tau j}=\frac{\theta_{\tau }\left(d_{j}\right)\left(|D|-1+e^{\varepsilon_{\tau }}\right)-n_{\tau }}{e^{\varepsilon_{\tau }}-1}$$

The true frequency distribution of the discretized boundary values in subgroup $G_{\tau }$ is defined as $\phi_{\tau }=\left\{\phi_{\tau 1},\phi_{\tau 2},\ldots ,\phi_{\tau k}\right\}$, and the calibrated estimated frequency distribution of the boundary values is defined as ${\overset{\wedge }{\phi }}_{\tau }=\{{\overset{\wedge }{\phi }}_{\tau 1},{\overset{\wedge }{\phi }}_{\tau 2},\ldots ,{\overset{\wedge }{\phi }}_{\tau k}\}$. This paper employs the mean square error (MSE) as the metric for evaluating the accuracy of frequency estimation. The AG analyzes $\phi_{\tau }$ and ${\overset{\wedge }{\phi }}_{\tau }$, and defines its mean square error as $mse_{\tau }$ given by

(6) $$\begin{array}{cc} mse_{\tau } & =E\left[{\left\|\phi_{\tau }-{\overset{\wedge }{\phi }}_{\tau }\right\|}_{2}^{2}\right]=E\left[\sum_{j=1}^{k}{\left({\overset{\wedge }{\phi }}_{\tau j}-\phi_{\tau j}\right)}^{2}\right]\\ & =\sum_{j=1}^{k}\mathrm{Var}\left[{\overset{\wedge }{\phi }}_{\tau j}-\phi_{\tau j}\right]\\ & =\frac{1}{n_{\tau }\cdot {\left(p_{\tau }-q_{\tau }\right)}^{2}}\\ & \;\;\times \sum_{j=1}^{k}\left[\phi_{\tau j}p_{\tau }+\left(n_{\tau }-\phi_{\tau j}\right)q_{\tau }\right]\left[n_{\tau }-\phi_{\tau j}p_{\tau }-\left(n_{\tau }-\phi_{\tau j}\right)q_{\tau }\right] \end{array}$$

Here, $E\left[\cdot \right]$ denotes the expectation operator, ${\left\|\cdot \right\|}_{2}^{2}$ represents the two-norm operation, and $\mathrm{Var}\left[\cdot \right]$ denotes the variance operator. From the above equation, the MSEs of the frequency estimate for all privacy groups $G_{1},G_{2},\ldots ,G_{m}$ can be obtained as $mse_{1},mse_{2},\ldots ,mse_{m}$. This metric quantifies the reliability of data from different subgroups and provides the foundation for subsequent weight assignment.

For the subgroup $G_{\tau }$, let the true local mean of its electricity consumption data be denoted as $\mu_{\tau }$, and the corresponding estimated local mean as ${\hat{\mu }}_{\tau }$ given by

(7) $${\hat{\mu }}_{\tau }=\frac{1}{n_{\tau }}\cdot \sum_{j=1}^{k}d_{j}\cdot {\overset{\wedge }{\phi }}_{\tau j}$$

Consequently, the estimated local means of electricity consumption data for the subgroups can be derived.

#### 4.4.2. MSE-Based Weighted Aggregation Strategy

In the EDAS-PLDP scheme, the selection of privacy protection levels inherently introduces a trade-off between privacy strength and data utility: subgroups with a small $\varepsilon_{\tau }$ (strong privacy) experience significant noise addition, leading to high estimation errors; conversely, subgroups with a large $\varepsilon_{\tau }$ (weak privacy) yield more accurate estimates but offer weaker privacy protection. This heterogeneity poses a challenge for accurate aggregation—if simple averaging is applied, the noise from strong-privacy subgroups will severely impact the overall aggregation accuracy. The MSE-based weighting strategy proposed in this paper is precisely designed to address this trade-off. By assigning lower weights to subgroups with high MSE, it moderately sacrifices the data contribution of highly protected users while ensuring overall analytical accuracy, thereby achieving a balance between individualized privacy requirements and the data utility required by the power grid. For the subgroup $G_{\tau }$, its weighting factor is defined as $w_{\tau }$:

(8) $$w_{\tau }=\frac{1/mse_{\tau }}{\sum_{i=1}^{m}1/mse_{i}}$$

Thus, the weight set for each subgroup is obtained as $w=\left\{w_{1},w_{2},\ldots ,w_{m}\right\}$, satisfying $\sum_{\tau =1}^{m}w_{\tau }=1$. This weighted aggregation strategy takes into account not only the number of end-users and the privacy budget in different privacy groups, but also incorporates the heterogeneity of data distributions across groups. As a result, the privacy group with a smaller MSE (i.e., lower $mse_{\tau }$) will be assigned a bigger weighting factor, while the other with a larger MSE (i.e., higher $mse_{\tau }$) will be assigned a smaller weighting factor. This approach enhances the contribution of subgroups with low estimation error and suppresses the influence of subgroups with high estimation error, thereby improving the overall aggregation accuracy of our proposed EDAS-PLDP scheme.

Let the global mean of the electricity consumption data across all subgroups be denoted as μ. The estimated global mean $\hat{\mu }$ is obtained via weighted aggregation as:

(9) $$\hat{\mu }=\sum_{i=1}^{m}w_{i}{\hat{\mu }}_{i}=\sum_{i=1}^{m}\sum_{j=1}^{k}\frac{w_{i}d_{j}{\overset{\wedge }{\phi }}_{ij}}{n_{i}}$$

**Lemma** **1.** *The estimated global mean* $\hat{\mu }$ *obtained through the weighted aggregation strategy is an unbiased estimator of the true global mean* μ*.*

**Proof** **of** **Lemma** **1.** From Equation (5), for each subgroup $G_{\tau }$, the estimated frequency ${\overset{\wedge }{\phi }}_{\tau j}$ satisfies $E[{\overset{\wedge }{\phi }}_{\tau j}]=\phi_{\tau j}$. Then the estimated local mean ${\hat{\mu }}_{\tau }$ satisfies:

(10) $$\begin{array}{cc} E[{\hat{\mu }}_{\tau }] & =\frac{1}{n_{\tau }}\sum_{j=1}^{k}d_{j}E[{\overset{\wedge }{\phi }}_{\tau j}]\\ & =\frac{1}{n_{\tau }}\sum_{j=1}^{k}d_{j}\phi_{\tau j}\\ & =\mu_{\tau } \end{array}$$

Therefore, the estimated local mean ${\hat{\mu }}_{\tau }$ is unbiased. Hence, the expectation of the estimated global mean satisfies:

(11) $$\begin{array}{cc} E[\hat{\mu }] & =E[\sum_{i=1}^{m}w_{i}{\hat{\mu }}_{i}]\\ & =\sum_{i=1}^{m}w_{i}E[{\hat{\mu }}_{i}]\\ & =\mu \sum_{i=1}^{m}w_{i}\\ & =\mu \end{array}$$

Then it has that $E[\hat{\mu }]=\mu$, so the weighted aggregation strategy is unbiased. □

## 5. Experimental Results and Comparative Analysis

To validate the effectiveness and efficiency of the proposed EDAS-PLDP scheme, we conducted comprehensive experiments under various scenarios. This section presents the experimental setup, followed by detailed comparative analyses on estimation accuracy, computational efficiency, and robustness against attacks.

### 5.1. Experimental Setup

The experimental platform employs an Intel Core i7-4770 CPU (3.40 GHz) with 16 GB of memory, running the Windows 10 operating system. All experiments were implemented using Python 3.9.0, with numpy version 1.15.1. Here, each experiment will run 100 times, and the results reported in the experiments are the averages of all runs. The estimation accuracy was evaluated using the MSE, where a smaller MSE indicates that the estimated value is closer to the true value, reflecting better performance:

(12) $$\mathrm{MSE}=\frac{1}{100}\sum_{iter=1}^{100}{\left(\mu -{\hat{\mu }}_{iter}\right)}^{2}$$

Here, ${\hat{\mu }}_{iter}$ denotes the global estimated mean obtained at each iteration.

To quantify the anti-attack performance advantages of the proposed EDAS-PLDP, the experiments introduce the Relative Error (RE) relative to comparative schemes as a quantitative metric.

(13) $$\mathrm{RE}=\frac{1}{100}\sum_{iter=1}^{100}\frac{\left|\mu -{\hat{\mu }}_{iter}^{\prime }\right|}{\mu }$$

Here, ${\hat{\mu }}_{iter}^{\prime }$ denotes the global estimated mean computed at each iteration under false data injection attacks.

This experiment aims to evaluate the performance of the proposed EDAS-PLDP under different conditions. We compare our scheme with Gai’s scheme and Li’s scheme. The validation of the scheme is conducted on the following two datasets: the Gaussian synthetic dataset and the UK-DALE real dataset. The UK-DALE dataset [24] is an open-source dataset from the UK that records the power demand of five houses with a collection accuracy of 6 s, and the longest household recorded 655 days.

We consider three subgroups $G_{1}$, $G_{2}$, $G_{3}$ (i.e., *m* = 3). In the simulation, we set three proportion scenarios of the three subgroups—$\alpha_{1}$, $\alpha_{2}$, $\alpha_{3}$. These scenarios are designed to capture common structural variations: $\alpha_{1}$ represents a distribution skewed towards the group with the weakest privacy protection, $\alpha_{2}$ models a balanced distribution, and $\alpha_{3}$ models a distribution skewed towards the group with the strongest privacy protection; the concrete parameter settings are listed in Table 1. Additionally, we simulate three privacy-preserving level scenarios for smart terminals—$\beta_{1}$, $\beta_{2}$, $\beta_{3}$—to simulate three personalized privacy protection demands; the detailed parameters are shown in Table 2.

The results presented in the next subsection will specifically illustrate the performance of EDAS-PLDP under these personalized privacy configurations, including its estimation accuracy variations across different scenarios, comparative advantages over baseline methods, and the effectiveness of the weighted aggregation strategy in balancing privacy protection and data utility.

### 5.2. Estimation Accuracy with Multiple Scenarios

**Figure 2:**
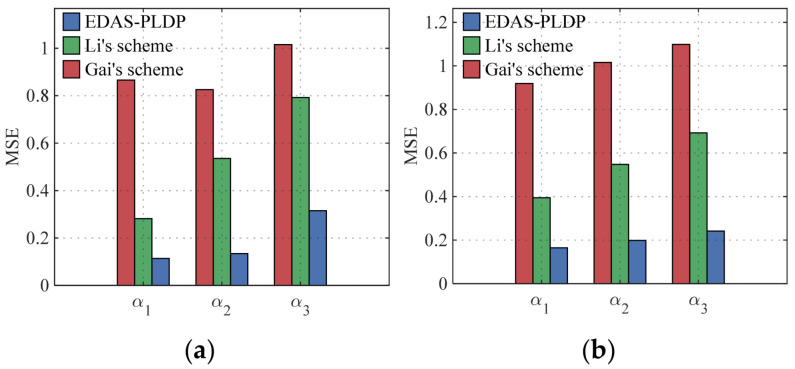
MSEs under three proportion settings: (**a**) Gaussian; (**b**) UK-DALE.

Figure 2 shows the MSEs under three proportion settings in two datasets, with the number of end-users set to n=50k, the number of electricity consumption subintervals set to d=10, and the privacy-preserving level scenario set to $\beta_{1}$. The simulation results indicate that, under the same conditions, the MSE of the proposed EDAS-PLDP is significantly lower than that of Li’s scheme and Gai’s scheme. For example, under the given condition $\alpha_{1}$ in the Gaussian dataset, the estimation accuracy of EDAS-PLDP improves by roughly 80% relative to Gai’s scheme and by about 60% relative to Li’s scheme. This advantage stems from the weighted aggregation strategy based on MSE, which greatly enhances statistical estimation accuracy. Although Li’s scheme supports personalized privacy protection, its weighted aggregation mechanism neglects heterogeneity in subgroup distributions; its weight allocation relies solely on a preset fixed variance, leading to accuracy loss.

**Figure 3:**
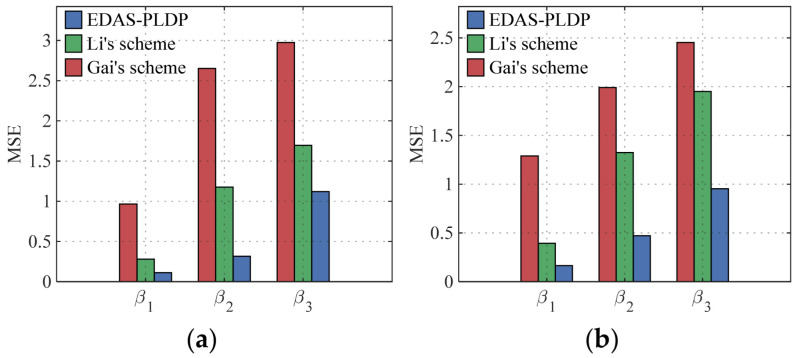
MSEs under three privacy-preserving level scenarios: (**a**) Gaussian; (**b**) UK-DALE.

Figure 3 presents the MSEs under three privacy-preserving level scenarios in two datasets, with the total end-users’ number set to n=50k, the number of electricity consumption subintervals set to d=10, and the proportion scenario set to $\alpha_{1}$. The results show that the MSE of our proposed EDAS-PLDP is markedly lower than that of the two baselines. For instance, under the specified condition $\beta_{1}$ in the Gaussian dataset, the MSE of our EDAS-PLDP scheme improves by roughly 63% relative to Gai’s scheme and roughly 34% relative to Li’s scheme. In summary, the significant aggregation accuracy advantage of our EDAS-PLDP scheme stems from the weighted aggregation strategy based on MSE.

The above experimental results confirm that EDAS-PLDP maintains high estimation accuracy across diverse privacy scenario settings. Beyond static scenario analysis, we next investigate how estimation accuracy scales with the number of end-users, which is a critical factor for practical deployment in smart grids.

### 5.3. Estimation Accuracy vs. the Number of End-Users

**Figure 4:**
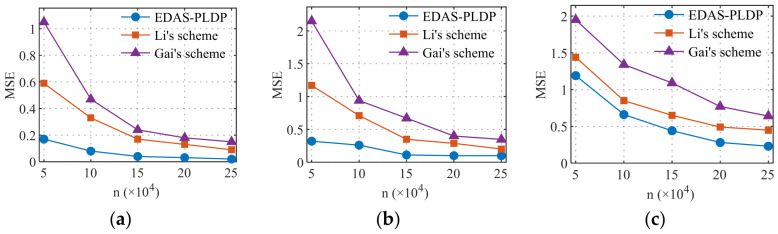
MSEs under different numbers of end-users (Gaussian): (**a**) $\left(\alpha_{1},\beta_{1}\right)$; (**b**) $\left(\alpha_{2},\beta_{2}\right)$; (**c**) $\left(\alpha_{3},\beta_{3}\right)$.

**Figure 5:**
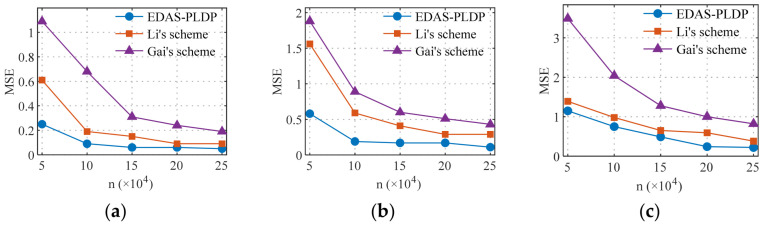
MSEs under different numbers of end-users (UK-DALE): (**a**) $\left(\alpha_{1},\beta_{1}\right)$; (**b**) $\left(\alpha_{2},\beta_{2}\right)$; (**c**) $\left(\alpha_{3},\beta_{3}\right)$.

Figure 4 and Figure 5 illustrate the influence of the number of end-users on the MSE in two datasets, respectively, where the number of electricity consumption subintervals was set as d=10, and the number of end-users n ranges from small to large-scale smart grid scenarios. The results show that our proposed EDAS-PLDP consistently demonstrates significant accuracy advantages compared with the existing two schemes. For example, under the case of $\left(\alpha_{1},\beta_{1}\right)$ in Gaussian dataset, the proposed EDAS-PLDP scheme improves performance by approximately 74% compared to Gai’s scheme and by about 63% compared to Li’s scheme. This advantage stems from the MSE-based weighted aggregation strategy employed in the EDAS-PLDP scheme, which adjusts the contribution of different privacy groups to the final aggregation result, mitigates noise accumulation effects, and maintains excellent data aggregation performance even in small-scale end-user scenarios.

After analyzing the impact of user population size, we will next investigate the effect of data discretization granularity, controlled by the number of electricity consumption subintervals, on aggregation accuracy.

### 5.4. Estimation Accuracy vs. the Number of Electricity Consumption Subintervals

**Figure 6:**
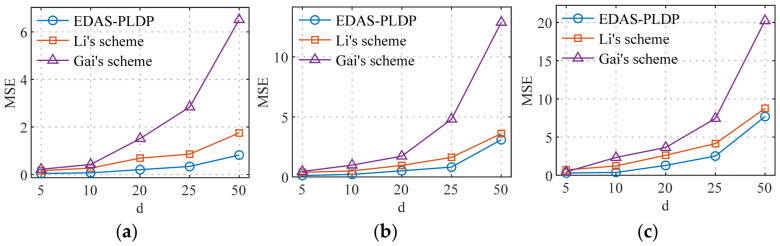
MSEs under different numbers of electricity consumption subintervals (Gaussian): (**a**) $\left(\alpha_{1},\beta_{1}\right)$; (**b**) $\left(\alpha_{2},\beta_{2}\right)$; (**c**) $\left(\alpha_{3},\beta_{3}\right)$.

**Figure 7:**
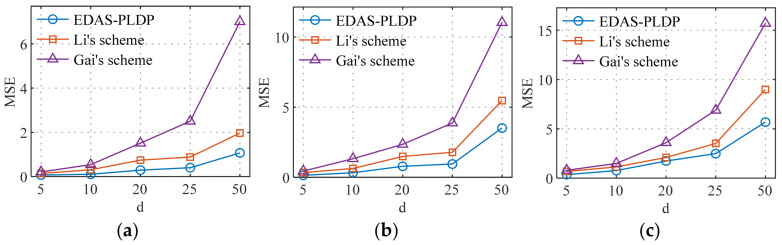
MSEs under different numbers of electricity consumption subintervals (UK-DALE): (**a**) $\left(\alpha_{1},\beta_{1}\right)$; (**b**) $\left(\alpha_{2},\beta_{2}\right)$; (**c**) $\left(\alpha_{3},\beta_{3}\right)$.

Figure 6 and Figure 7, respectively, simulate the influence of subintervals on the MSE in two datasets, with the total number of end-users set to n=100k. The number of electricity consumption subintervals was set to 5, 10, 15, 20, and 25, covering scenarios ranging from coarse-grained to fine-grained partitioning of electricity consumption data. The experimental results indicate that as the number of the subintervals d increases, the MSEs of all three schemes show a rising trend, and the error amplification effect becomes more pronounced with larger d. This phenomenon can be attributed to the increased data sparsity caused by overly fine subinterval divisions, which raises the difficulty of correcting data after randomized response perturbation, thereby reducing aggregation accuracy. Moreover, under the same conditions, our proposed EDAS-PLDP scheme demonstrates the best aggregation accuracy compared to the two baseline schemes. This advantage also stems from the ability of EDAS-PLDP to adjust the weighting factors of different subgroups based on MSE.

Beyond accuracy considerations, the computational efficiency of a privacy-preserving scheme is equally crucial for practical deployment in resource-constrained smart grid environments. The following subsection presents a comprehensive efficiency analysis of EDAS-PLDP from both SM and AG perspectives.

### 5.5. Efficiency Analysis

To comprehensively evaluate the practicality of the EDAS-PLDP scheme in real-world deployment, this section conducts a comparative analysis of computational efficiency from both the SM and AG perspectives. Computational overhead is a key metric for assessing whether the scheme can operate efficiently in the resource-constrained environment of smart grids. The experimental setup is as follows: the number of sub-intervals is set to d=10, the proportion scenario is set to $\alpha_{1}$, and the privacy-preserving level scenario set to $\beta_{1}$. The efficiency of the three methods is tested under various scales, including million-level end-user scenarios typical of smart grids.

**Figure 8:**
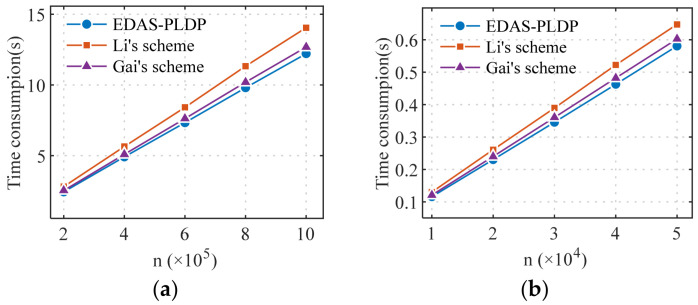
Computational efficiency comparison of SM: (**a**) Gaussian; (**b**) UK-DALE.

As end-side devices, SM typically possess limited computational and memory resources. Figure 8 shows the comparison of average time consumption on the SM side for the three schemes. The proposed EDAS-PLDP scheme achieves the best computational efficiency at the SM side; the time required for data discretization and perturbation is less than $1.3\times 10^{-5}$ s, demonstrating that such computational tasks are well within the capabilities of resource-constrained SM. Although it requires independent processing for user groups with different privacy levels, since Gai’s scheme employs the minimum privacy budget required to satisfy the privacy protection needs of all users, the resulting larger perturbation amplitude leads to a longer runtime compared to the scheme proposed in this paper. The Li’s scheme, which needs to support multiple randomized-response mechanisms, incurs the highest time cost.

**Figure 9:**
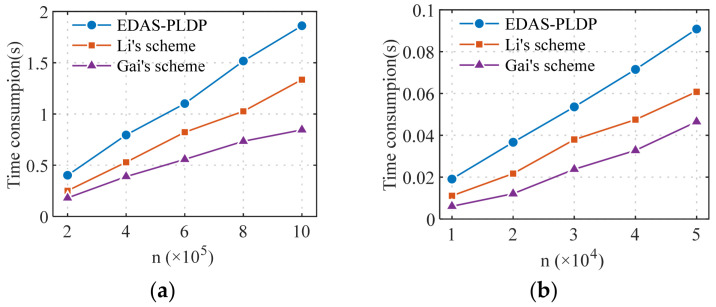
Computational efficiency comparison of AG: (**a**) Gaussian; (**b**) UK-DALE.

Figure 9 presents a comparison of computational efficiency on the AG side for the three schemes. The experimental results indicate that the EDAS-PLDP scheme incurs the highest computational overhead at the AG side, primarily due to the MSE-based weighted aggregation strategy introduced during the aggregation phase. Although these operations increase the computational burden, they significantly improve aggregation accuracy. As shown in Figure 9a, the AG in the proposed EDAS-PLDP scheme completes the aggregation task for electricity consumption data from millions of end users in less than 2 s, meeting the practical requirements for gateway processing efficiency in real-world deployment. The Li’s scheme also exhibits higher overhead than the Gai’s scheme due to its complex perturbation mechanisms.

Overall, the computational efficiency of the EDAS-PLDP scheme is comparable to that of Gai’s scheme, while the latter lacks personalized privacy protection. Although the Li’s scheme supports personalized privacy, its computational overhead is the highest, making it difficult to deploy in large-scale networks. Compared with the Li’s scheme, the proposed EDAS-PLDP scheme achieves significantly lower computational overhead while substantially improving aggregation accuracy. This balance makes EDAS-PLDP more suitable for large-scale smart grid scenarios (such as those with millions of end users) where both data accuracy and personalized privacy protection are critical requirements.

### 5.6. Comparative Analysis of Resilience to False Data Injection Attacks

To comprehensively evaluate the robustness of the proposed EDAS-PLDP scheme under false data injection attack scenarios, this experiment designs targeted anti-attack simulations to test its performance under varying levels of attacks. The attack proportions are set to: $\rho_{1}=5\%,\rho_{2}=10\%,\rho_{3}=20\%$. Setting the number of end-users to 10,000, the privacy configuration is set to $\left(\alpha_{1},\beta_{3}\right)$. It is assumed that the actual electricity consumption data is x, and false data are randomly generated from the interval $\left[0.5x,1.5x\right]$ to replace the original real consumption data. This simulates attackers simultaneously injecting abnormally low or high electricity values, thereby interfering with the normal monitoring and dispatch operations of the power grid.

**Figure 10:**
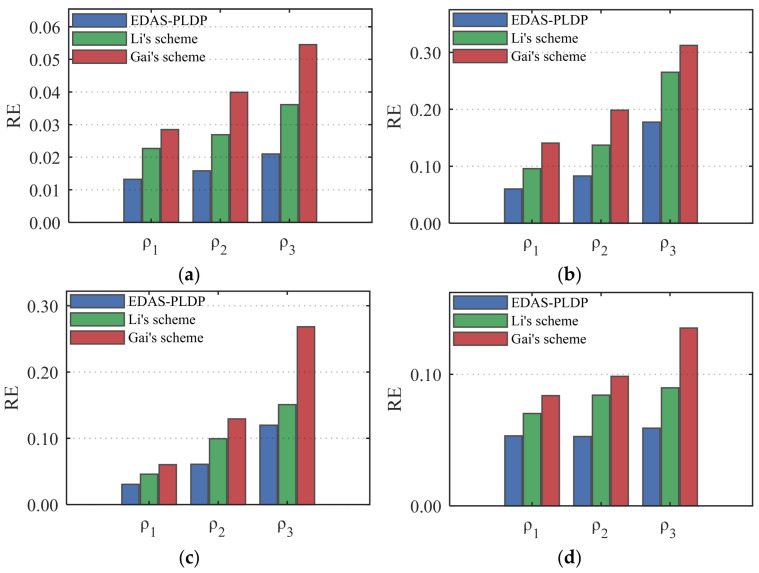
The impact of injection attack ratio on RE: (**a**) Gaussian; (**b**) UK-DALE; (**c**) Uniform; (**d**) AMPds.

In this subsection, to further validate the effectiveness and feasibility of the experiment, we also conduct experimental comparisons in the Uniform synthetic dataset and the real-world AMPds dataset [25]. Figure 10 shows that the proposed EDAS-PLDP scheme exhibits significant resilience against false data injection attacks across various injection ratios. For instance, on the UK-DALE dataset, its overall anti-attack performance improved by 36% and 52% compared with Li’s scheme and Gai’s scheme, respectively. This advantage primarily stems from the MSE-based weighted aggregation strategy deployed at the gateway side, which dynamically assesses the estimation reliability of each privacy group and automatically reduces the aggregation weight of groups that are under attack or exhibit higher noise levels. Consequently, the impact of false electricity-consumption data on the final estimation result is suppressed at the global level. Thus, even in the presence of false data injection attacks, EDAS-PLDP maintains more reliable data aggregation quality than both Li’s scheme and Gai’s scheme.

## 6. Discussion

The comprehensive experimental evaluation substantiates that the proposed EDAS-PLDP scheme consistently and significantly outperforms state-of-the-art benchmarks in three critical dimensions: estimation accuracy, computational efficiency, and attack resilience. These compelling results provide direct empirical validation for our core thesis: by innovatively integrating a personalized privacy protection framework with a credibility-aware aggregation strategy based on MSE, we successfully resolve the fundamental tension between personalized privacy protection and high data utility in smart grids, thereby establishing a new effective balance for smart grid applications.

Compared to Gai et al.’s scheme, our method achieves significantly lower MSE, especially when end-users exhibit diverse privacy preferences. This improvement stems from EDAS-PLDP’s ability to adaptively weight subgroups based on estimation reliability, reducing the influence of noisy or low-utility data. Furthermore, unlike Li et al.’s personalized scheme, our approach maintains competitive accuracy with lower computational overhead, making it more suitable for large-scale smart grid deployments. The proposed EDAS-PLDP also demonstrates robustness against false data injection attacks, leveraging distributed perturbation and MSE-based credibility evaluation to ensure result integrity even under adversarial conditions. From a broader perspective, this work contributes to privacy-aware data analytics in cyber-physical systems and can be extended to domains such as healthcare data sharing and the industrial Internet of Things. Although simulation experiments have validated the effectiveness of EDAS-PLDP in controlled environments, several practical challenges must be addressed to achieve its large-scale deployment in real-world smart grids:

1. Hardware resource constraints: Although the current scheme achieves extremely low terminal computational overhead in simulations, smart meters in practical deployment are often resource-constrained devices, which may require additional consideration for energy optimization. Future work needs to explore more lightweight implementations of meter computations and conduct performance tests on typical hardware platforms to ensure the compatibility and stability of the scheme on both existing and new devices.
2. Network environment uncertainty: Real-world power grids rely on communication networks for data transmission and may face issues such as packet loss, latency fluctuations, and bandwidth limitations. If some users’ uploaded data is lost due to network failures, the gateway side will face incomplete datasets. Future research needs to investigate aggregation mechanisms with fault tolerance, such as missing value compensation based on historical data or distributed caching strategies, to ensure estimation accuracy under non-ideal communication conditions.

## 7. Conclusions

Facing the contradiction between personalized privacy protection and high-precision data utility in smart grids, this paper proposes an efficient data aggregation scheme based on personalized local differential privacy (EDAS-PLDP). The scheme allows users to autonomously select their privacy protection levels, breaking through the traditional “one-size-fits-all” model, and innovatively introduces a mean square error-based weighted aggregation strategy at the gateway side. By evaluating the data reliability of different privacy groups to assign weights, it amplifies the contribution of high-quality data while suppressing noise from strong privacy perturbations. Extensive simulations demonstrate that EDAS-PLDP consistently outperforms existing baseline schemes in estimation accuracy across various privacy preference distributions, user scales, and data granularities. Moreover, the scheme maintains competitive computational efficiency with lower overhead compared to other personalized approaches, enhancing its feasibility for large-scale deployment. Its inherent distributed perturbation architecture, coupled with the MSE-based weighted aggregation strategy, also endows EDAS-PLDP with significant resilience against false data injection attacks. This scheme provides a practical solution for balancing personalized privacy protection and data utility in smart grids.

This study opens up several promising directions worth exploring in the field of smart grid privacy protection. First, the selection mechanism for end-users’ privacy protection levels deserves further investigation—adjusting privacy protection strength in real time by sensing changes in user behavior and grid operating conditions to optimize privacy and utility. Second, with the development of edge intelligence, future work could explore combining EDAS-PLDP with lightweight federated learning to support more complex grid analysis tasks, such as anomaly detection and load forecasting, while ensuring personalized privacy protection. The flexible methodological framework it provides can also be adapted and extended to other data-sensitive physical systems, such as smart healthcare and the industrial Internet of Things, where the dual imperatives of individual privacy and collective data utility must be strategically reconciled.

## Figures and Tables

**Table 1:** Three proportion scenarios.

Proportion Scenario	Parameter Settings
$\alpha_{1}$	$n_{1}:n_{2}:n_{3}=2:3:5$
$\alpha_{2}$	$n_{1}:n_{2}:n_{3}=4:2:4$
$\alpha_{3}$	$n_{1}:n_{2}:n_{3}=5:3:2$

**Table 2:** Three privacy-preserving level scenarios.

Privacy-Preserving Level Scenario	Parameter Settings
$\beta_{1}$	$\varepsilon_{1}=1.0,\varepsilon_{2}=1.5,\varepsilon_{3}=2.0$
$\beta_{2}$	$\varepsilon_{1}=0.8,\varepsilon_{2}=1.2,\varepsilon_{3}=1.6$
$\beta_{3}$	$\varepsilon_{1}=0.7,\varepsilon_{2}=1.0,\varepsilon_{3}=1.3$

## Data Availability

The original contributions presented in this study are included in the article. Further inquiries can be directed to the corresponding author.
